# Development of a mortality prognostic model based on the Pneumonia Severity Index for older adults with pneumonia in integrated medical and older adult care institutions: a retrospective study

**DOI:** 10.3389/fmed.2026.1913119

**Published:** 2026-10-30

**Authors:** Xiaoshan Du, Yinglin Wu, Shengkui Wang, Gang Ni

**Affiliations:** Geriatrics Department, The Third Affiliated Hospital of Anhui Medical University (The First People’s Hospital of Hefei), Hefei, Anhui, China

**Keywords:** CFS score, MDRO infection, PNI, PSI score, risk factors, ROC curve

## Abstract

**Background:**

Population aging has increased the demand for integrated medical and long-term care services, particularly in China. Age-related respiratory impairment and immunosenescence render institutionalized older adults at a high risk for pneumonia-associated with poorer outcomes than community-acquired pneumonia. We aimed to investigate the prognostic value of the Pneumonia Severity Index (PSI) for mortality in older adults with pneumonia in integrated medical and elderly care facilities, and to develop a prognostic prediction model.

**Methods:**

Medical records of older adult patients with pneumonia admitted to the integrated medical-elderly care ward of the Department of Geriatrics, Hefei First People's Hospital from June 2019 to June 2024 were retrospectively retrieved. A total of 312 patients were enrolled according to predefined inclusion and exclusion criteria. Patients were divided into a survival group (*n* = 232) and a non-survival group (*n* = 80) based on post-treatment clinical outcomes. Univariate analyses were performed to compare clinical characteristics between the two groups. All clinical variables were screened using Least Absolute Shrinkage and Selection Operator (LASSO) regression. Variables with non-zero coefficients were subsequently incorporated into Firth's bias-corrected logistic regression to construct the prediction model, followed by model validation.

**Results:**

Univariate analysis revealed significant intergroup differences in age ≥80 years, coronary heart disease, white blood cell count (WBC),neutrophil count, multidrug-resistant organism (MDRO) infection, PSI score, hemoglobin, creatinine, coronavirus disease 2019 (COVID-19)infection, Clinical Frailty Scale (CFS) score >7, Prognostic Nutritional Index (PNI) < 35, and C-reactive protein (CRP) (all *P* ≤ 0.05). LASSO regression with *λ* = 0.0678392 identified six non-zero predictor variables: White blood cell count, MDRO infection, PSI score, CFS score >7, PNI <35, and CRP. A nomogram was developed using the coefficients obtained from Firth's bias-corrected logistic regression. The ROC analysis yielded an AUC of 0.889, sensitivity of 80%, specificity of 89%,and optimal cutoff value of 0.719. The model exhibited significantly better discriminative performance than PSI alone. Internal validation with 1000 bootstrap resamples produced a corrected AUC of 0.878, calibration slope of 0.9316, and intercept of −0.0584. Decision curve analysis (DCA) showed substantial net benefit within the threshold probability range of 0–0.6. The calibration curve demonstrated good agreement between predicted and observed mortality risks of the nomogram.

**Conclusion:**

PSI is an independent risk factor for mortality among older adults with pneumonia in integrated medical and elderly care facilities. The model combining PSI with white blood cell count, MDRO infection, CFS score >7, PNI <35, and CRP can effectively predict mortality risk in this population and has superior clinical utility compared with PSI alone.

## Introduction

The increase in the aging population globally has prompted many countries to implement policies that address related demographic challenges. In the unique social and economic context of China, an integrated medical and older adult care service model has been developed, strongly supported by the government (1). Older adults often suffer from geriatric syndromes, such as frailty, malnutrition, sarcopenia, and dementia,leading to loss of independence and long-term care needs in such institutions (2). Age-related respiratory changes, including a weakened cough reflex, reduced chest wall mobility, decreased lung compliance, and impaired mucociliary clearance,compromise the airway defense mechanisms (3). Further, advanced age, declining immunity, and organ senescence increase the risk of pneumonia in institutionalized older adults (4). The prognosis of pneumonia acquired in integrated care settings is substantially worse than that of community-acquired pneumonia (5). Pneumonia exacerbates underlying comorbidities, prolongs hospital stay, increases medical costs (6), and is the leading cause of death in older adults (7).

Independent risk factors for mortality in older adults with pneumonia include demographic characteristics, comorbidities, frailty and nutritional status, vital signs, pneumonia severity scores, laboratory inflammatory markers, and pathogen-related factors (8–17). Demographic risk factors comprise advanced age, male sex, and long-term residence in nursing facilities (9–11). Underlying comorbidities such as coronary artery disease,chronic obstructive pulmonary disease, cerebral infarction, and renal failure may deteriorate during acute pneumonia, potentially leading to multiple organ failure and death (12). Frailty and malnutrition are common geriatric syndromes that reflect diminished physiological reserve and reduced stress tolerance. Their combined effects contribute to poor treatment response to pneumonia and elevate the risk of progression to respiratory failure and sepsis (13, 14). Vital signs including blood pressure, respiratory rate, oxygen saturation, and body temperature serve as critical indicators for assessing pneumonia severity (15). Pneumonia severity scores can guide hospital admission decisions among patients with community-acquired pneumonia (16). Laboratory parameters such as white blood cell count, procalcitonin, albumin, blood urea nitrogen, and C-reactive protein are valuable for predicting pneumonia risk and prognosis (17). Infections caused by specific pathogens, including multidrug-resistant organisms and severe acute respiratory syndrome coronavirus 2 (SARS-CoV-2), are less responsive to conventional antimicrobial therapy and increase mortality among older patients with pneumonia (18).

Common pneumonia assessment scales include the Clinical Pulmonary Infection Score (CPIS), CURB-65 score, and Pneumonia Severity Index (PSI). The CPIS is primarily applied to adult ICU patients receiving mechanical ventilation for more than 48 h, with its core function to aid the diagnosis of ventilator-associated pneumonia (VAP). A CPIS score ≥6 indicates a high probability of VAP. Nevertheless, it serves only as an auxiliary reference and cannot be independently used for formal mortality prediction, and is not recognized as a validated prognostic scoring tool (19). The CURB-65 score is designed for adult community-acquired pneumonia (CAP) and widely adopted in older adults with CAP, yet it has limited performance in dynamically monitoring treatment response and estimating mortality (20). The PSI score is also developed for adult CAP to predict 30-day mortality and guide site-of-care decisions. Its variables incorporate age, underlying comorbidities, and systemic laboratory parameters. As the most widely used scale for pneumonia risk stratification and clinical mortality prediction, the PSI covers demographic factors, baseline comorbidities, vital signs, laboratory tests, and imaging findings (Table 1) (21). Pneumonia among older adults in integrated medical and elderly care facilities is classified as hospital-acquired pneumonia. Inpatient cases in this population are characterized by severe frailty, compromised nutritional and immune status, susceptibility to multidrug-resistant pathogens, and high risk of clinical deterioration. Accordingly, the PSI has certain limitations when evaluating this specific pneumonia subgroup, and its prognostic performance for older adults with pneumonia remains unclear (22). This study aimed to explore the prognostic value of the PSI for mortality in older patients with pneumonia from integrated medical and elderly care facilities, screen high-risk factors for death, and develop a combined prognostic prediction model, so as to provide evidence for clinical management and decision-making. The results are reported below.

**Table 1:** Pneumonia severity index (PSI) scoring scale.

Scoring items	Points	Scoring items	Points	Scoring items	Points
Demographic Factors		Comorbid Illnesses		Physical Examination Findings	
Male (Age value)	Age	Neoplastic disease	30	Altered mental status	20
Female (Age value)	Age−10	Liver disease	20	Respiratory rate ≥ 30 breaths/min	20
Nursing home resident	10	Congestive heart failure	10	Systolic BP < 90 mmHg	20
Laboratory & Imaging Findings		Cerebrovascular disease	10	Temperature < 35 °C or ≥ 40 °C	15
Arterial pH < 7.35	30	Renal disease	10	Pulse ≥ 125 beats/min	10
BUN ≥ 30 mg/dL	20	Serum sodium < 130 mEq/L	20	Glucose ≥ 250 mg/dL	10
Hematocrit < 30%	10	PaO₂ < 60 mmHg/SpO₂ < 90%	10	Pleural effusion	10

Note: The pneumonia severity index (PSI) was developed for mortality risk stratification in patients with community-acquired pneumonia. Adapted from Fine et al. (21).

## Material and methods

### Study population

In this retrospective study, we reviewed the data of older patients receiving long-term care in the integrated medical and older adult care ward of the Southern Campus of Hefei First People's Hospital between June 2019 and June 2024. Overall, 312 older patients with pneumonia in medical–nursing combined institutions were included and categorized into survival (*n* = 232) and non-survival (*n* = 80) groups according to their prognoses. The inclusion criteria were as follows: age ≥65 years; meeting the diagnostic criteria for pneumonia⁸; having received standard treatment for pneumonia, including antimicrobial therapy, expectorant management, assisted turning and back percussion, and oxygen therapy or airway humidification as needed; and having complete clinical data. The exclusion criteria were as follows: discharge against medical advice before treatment completion; coexisting primary immunodeficiency; and secondary infection from other organ systems. The data selection process is shown in Figure 1.

**Figure 1:**
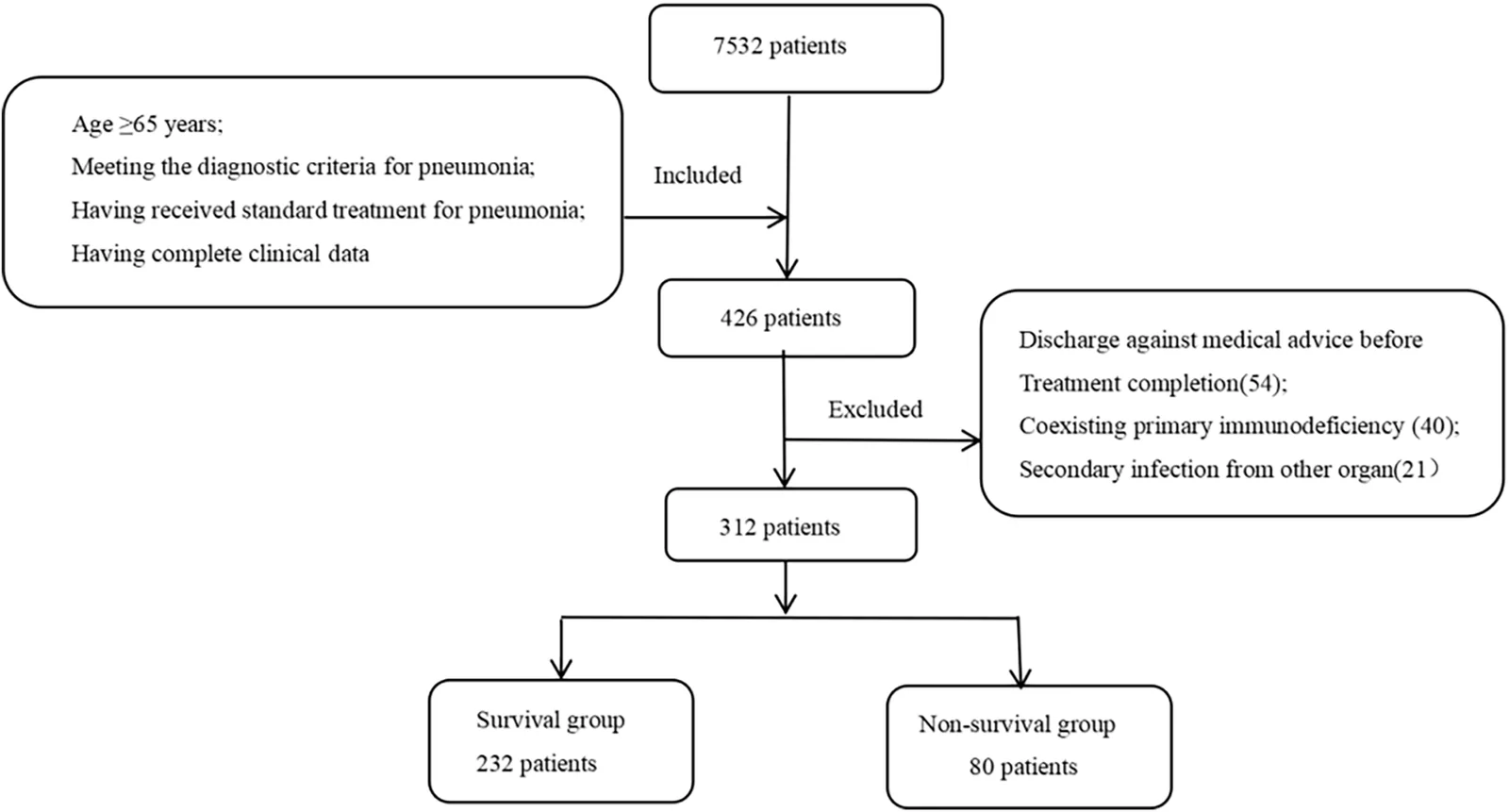
The ﬂowchart of patient selection.

### Diagnostic criteria

The included patients met the diagnostic criteria of the treatment guidelines for nursing home-acquired pneumonia based on communitys invol practice (8). A clinical diagnosis involves meeting at least two of the following items:

- Acute respiratory symptoms: new or aggravated cough, sputum production, dyspnea, and chest pain.
- Systemic infectious manifestations: fever (body temperature ≥ 38 °C) or hypothermia (body temperature < 36 °C), chills, and fatigue
- Pulmonary physical signs: moist rales, bronchial breath sounds, and pulmonary consolidation signs.
- Laboratory indicators: white blood cell (WBC) count > 10 × 10⁹/L or < 4 × 10⁹/L and elevated C-reactive protein (CRP)/procalcitonin (PCT) levels
- Atypical manifestations in older adults (available for independent diagnosis): confusion/drowsiness,functional decline,anorexia/decreased oral intake,dehydration, recurrent falls, and absence of fever and obvious cough.

### Sample size calculation

Sample size was calculated *a priori* using the method for multivariable prediction models proposed by Riley et al, implemented via the R package pmsampsize (20). The outcome was binary mortality. We prespecified a prediction model including six predictors, with an expected Cox-Snell R-squared (csrsquared) of 0.20 and an anticipated mortality rate of 25.6% in the study population. The calculation yielded a minimum required total sample size of 293 participants, with 76 mortality events. A total of 312 patients were finally enrolled in this study, with 80 mortality events. Both the sample size and event count met the requirements for model development.

### Missing data handling

Complete case analysis was adopted. Only samples with no missing values across all predictors and the outcome variable were retained for model development; cases with missing variables were excluded directly. This strategy was chosen for the following reasons: the overall missing rate was low, and the missing pattern approximated random. Multiple imputation was not performed to simplify model interpretation (21).

### Data collection

This was a retrospective study, and all clinical data were extracted from the electronic medical record system of our hospital. Candidate variables were predefined based on clinical expertise and previous literature on pneumonia prognosis. A data extraction form was developed in advance to collect demographic characteristics, comorbidities, vital signs, imaging findings, laboratory tests, infection-related indicators, and outcome variables. The PSI was calculated *post hoc* from the extracted clinical variables in accordance with the scoring criteria described in the original publication. Collected variables included baseline information (age, sex, etc.), underlying diseases (hypertension, diabetes mellitus, coronary artery disease, etc.), laboratory parameters (white blood cell count, red blood cell count, hematocrit, D-dimer, PNI, CRP, etc.), and clinical scores (PSI score, Full Outline of Unresponsiveness (FOUR) score, CFS score, etc.),Etiology [multidrug-resistant organism(MDRO) infection, coronavirus disease 2019 (COVID-19) infection] was analyzed to explore potential risk factors associated with prognosis.

### Statistical analysis

Statistical analyses were performed using SPSS 26.0. Normally distributed continuous variables were expressed as mean ± standard deviation and compared between groups using the independent-samples t test. Non-normally distributed continuous variables were presented as median (interquartile range) [M (Q1, Q3)], and between-group comparisons were conducted with the nonparametric Mann–Whitney U test. Categorical variables were reported as percentages and compared using the *χ*^2^ test. Lasso regression was used for initial screening of clinical candidate predictors. Variables with non-zero coefficients after screening were incorporated into Firth bias-corrected logistic regression, and the resulting regression coefficients were used to construct a multivariable prognostic nomogram. Receiver operating characteristic (ROC) curves of the nomogram and the standalone PSI were plotted simultaneously to compare their discriminative performance. Internal validation of the model was performed via Bootstrap resampling to calculate the optimism-corrected area under the curve (AUC). Calibration curves were adopted to assess the agreement between predicted risks and observed outcomes, and decision curve analysis (DCA) was applied to evaluate the clinical net benefit of the model. The development, validation, and reporting of this prediction model followed the TRIPOD statement (Type 1) (22).

## Results

### Comparison of baseline characteristics between the two groups

A total of 312 participants were enrolled in this study, including 80 non-survivors and 232 survivors. Baseline characteristics of all participants were presented, and univariable analysis was performed to compare baseline features between the non-survivor group and survivor group (Table 1). No statistically significant between-group differences were observed for sex, hypertension, diabetes mellitus, chronic obstructive pulmonary disease, platelet count, aspartate aminotransferase, alanine aminotransferase, serum potassium, D-dimer, tracheotomy, indwelling gastric tube, red blood cell count, monocyte count and Full Outline of Unresponsiveness (FOUR) score (*P* > 0.05)). Statistically significant differences [(*P* ≤ 0.05)] between non-survivors and survivors were identified for the following variables: multidrug-resistant organism (MDRO) infection, haemoglobin, Clinical Frailty Scale (CFS) > 7, coronary artery disease, white blood cell count, lymphocyte count, Pneumonia Severity Index (PSI) score, Prognostic Nutritional Index (PNI) < 35, creatinine, coronavirus disease 2019 (COVID-19), age ≥80 years, and C-reactive protein (CRP) (Table 2).

**Table 2:** Baseline characteristics.

Variables	Survival group (*n* = 232)	Non-survival group (*n* = 80)	*χ*2/t/Z	*P*
Age, *n* (%)			3.8391	0.05**
≥80 years	169 (72.84)	67 (83.75)		
<80 years	63 (27.16)	13 (16.25)		
Sex, *n* (%)			1.795	0.18
Male	125 (53.88)	50 (62.50)		
Female	107 (46.12)	30 (37.50)		
Hypertension, *n* (%)			3.038	0.081
Yes	119 (51.29)	32 (40.0)		
No	113 (48.71)	48 (60.0)		
Diabetes mellitus, *n* (%)			0.087	0.768
Yes	53 (22.84)	17 (21.25)		
No	179 (77.16)	63 (78.75)		
Coronary artery disease (CAD)，n (%)			8.914	0.003**
Yes	103 (44.40)	51 (63.75)		
No	129 (55.60)	29 (36.25)		
Chronic obstructive pulmonary disease, *n* (%)			1.252	0.263
Yes	29 (12.5)	14 (17.5)		
No	203 (87.5)	66 (82.50)		
Post-tracheostomy, *n* (%)			0.007	0.935
Yes	21 (9.05)	7 (8.75)		
No	211 (90.95)	73 (91.25)		
Indwelling nasogastric tube, *n* (%)			0.228	0.633
Yes	198 (85.34)	70 (87.5)		
No	34 (14.66)	10 (12.5)		
White Blood Cell Count (WBC)(×10⁹/L)	10.04 [6.82, 12.00]	15.30 [9.62, 19.99]	−6.214	0.000**
Neutrophil count (×10⁹/L)	14.93[4.93,11.61]	13.42[8.26,18.29]	−4.315	0.000**
Red blood cell count (×10^12^/L)	4.34 [3.17, 4.11]	4.81 [2.94, 4.03]	−0.699	0.484
Monocyte count(×10^12^/L)	0.76[0.37,0.66]	0.75[0.31,0.75]	−0.576	0.565
Lymphocyte count(×10^12^/L)	1.36[0.78,1.73]	1.09[0.54,1.45]	−2.902	0.004**
C-reactive protein(mg/L)	32.58[10.13,45.31]	101.13[39.29,142.32]	−8.56	0.001**
Platelet count (×10⁹/L)	232.86 [169.55, 288.75]	222.63[138.25,306.75]	−1.224	0.221
Hemoglobin level (g/L)	108.76 ± 1.43	102.44 ± 2.82	2.145	0.033**
Aspartate Transaminase level (U/L)	37.04 [21.00, 39.00]	36.30 [19.75, 41.00]	−0.17	0.865
Alanine Aminotransferase level (U/L)	32.48[15.00,32.75]	25.06[13.00,29.00]	−1.216	0.224
Serum Potassium concentration (mmol/L)	4.52 [3.76, 4.52]	4.37 [3.81, 4.86]	−1.852	0.064
D-Dimer (ng/L)	2.71[0.74, 2.41]	3.15 [3.62, 3.88]	−0.132	0.895
FOUR score, *n* (%)			1.271	0.259
≤8	130 (56.03)	39 (48.75)		
>8	102 (43.97)	41 (51.25)		
MDRO infection, *n* (%)			22.952	0.000**
Yes	42 (18.10)	36 (45)		
No	190 (81.90)	44 (55)		
COVID-19, *n* (%)			5.704	0.017**
Yes	11 (4.74)	10 (12.5)		
No	221 (95.26)	70 (87.5)		
CFS score, *n* (%)			26.751	0.001**
≤8	77 (33.19)	53 (66.25)		
>8	155 (66.81)	27 (33.75)		
PNI, *n* (%)			34.873	0.001**
＜35	183 (78.88)	35 (43.75)		
≥35	49 (21.12)	45 (56.25)		
PSI	166 [143, 187]	189.33[167,212]	−5.32	0.001**

MDRO, multi-drug resistant organism; PSI, pneumonia severity index; PNI, prognostic nutritional index; CFS, clinical frailty scale; COVID-19, Coronavirus Disease 2019; FOUR, full outline of unresponsiveness.

**Correlation is significant at *P* ≤ 0.05 level.

### Variable screening for mortality prognosis in the study sample

Variables listed in Table 2 were entered as independent variables into the Lasso regression analysis. Six variables with non-zero coefficients were selected (*λ* = 0.0678392), including PNI < 35, CFS > 7, PSI score, white blood cell count, multidrug-resistant organism (MDRO) infection, and C-reactive protein (CRP) (Figure 2).

**Figure 2:**
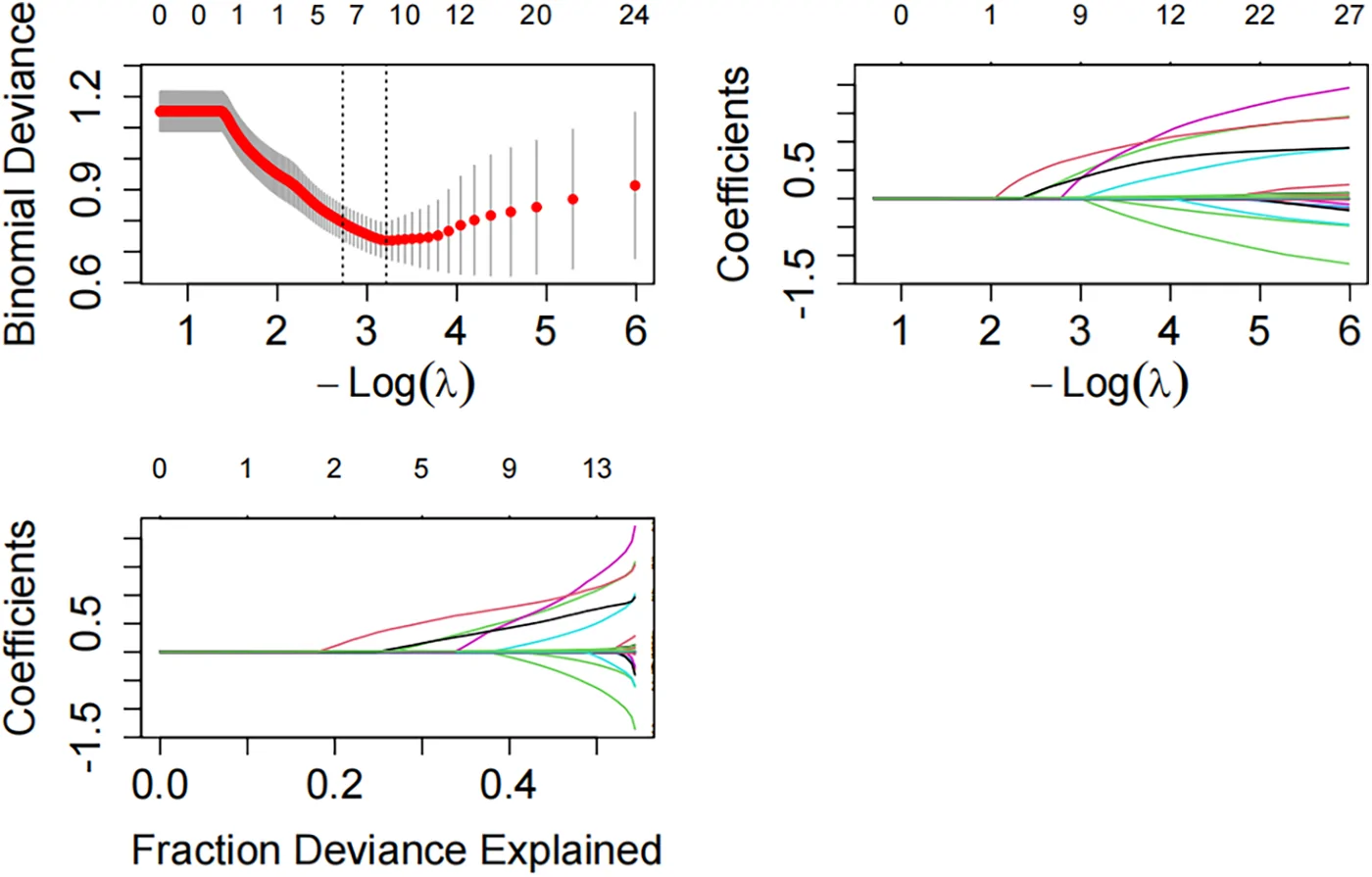
LASSO regression screening of variables associated with mortality prognosis among older patients with pneumonia in integrated medical and elderly care facilities.

### Firth bias-corrected logistic regression analysis of the study sample

Variables selected Variables selected by Lasso regression were further subjected to Firth bias-corrected logistic regression analysis. The results showed that PNI < 35, CFS > 7, PSI score, multidrug-resistant organism (MDRO) infection, and C-reactive protein (CRP) were independent predictors of mortality (*P* ≤ 0.05). White blood cell (WBC) count (*P* = 0.058, 95%CI:1.000–1.109), although selected by Lasso regression, exhibited potential predictive value and was therefore retained in the final prediction model. The prediction model was established based on the following formula: *P* = 1/[1 + exp(−Z)]. The regression equation was as follows: Z = −6.2025 + 1.2914 × MDRO Infection+0.7948 × (CFS＞7) + 1.4265 × (PNI＜35) + 0.0532 × WBC Count+0.0102 × PSI+0.0250 × CRP. (Table 3).

**Table 3:** Firth bias-corrected logistic regression analysis of predictors for mortality in older patients with pneumonia in integrated medical and elderly care facilities.

Variables	B	SE	P	OR	95%CI
MDRO Infection	1.2914	0.3854	＜0.001	3.638	1.706–7.907
CFS＞7	0.7948	0.3633	0.029	2.214	1.085–4.644
PNI＜35	1.4265	0.3656	＜0.001	4.164	2.032–8.160
WBC Count	0.0532	0.0277	0.058	1.055	1.000–1.109
PSI	0.0102	0.0051	0.045	1.010	1.000–1.020
CRP	0.0250	0.0040	＜0.001	1.025	1.017–1.034
Constant	−6.2025	1.0030	＜0.001		

MDRO, multi-drug resistant organism; PSI, pneumonia severity index; PNI, prognostic nutritional index; CFS, clinical frailty scale; WBC, white blood cell; CRP, C-reactive protein; OR, odds ratio; CI, confidence interval.

### Construction of a nomogram for predicting mortality among older patients with pneumonia in integrated medical and elderly care facilities

A nomogram was developed based on the Firth-adjusted regression coefficients using the variables selected by Lasso regression: PNI < 35, CFS > 7, PSI score, WBC count, MDRO infection, and CRP. For each patient, the point score for each predictor was read from the corresponding variable scale. The individual point scores of all variables were summed to obtain the total points. The total points were then mapped to the linear predictor, which was further converted to the predicted probability of adverse outcome (Risk). The probability corresponding to the total points represented the predicted mortality risk of pneumonia for older adults in integrated medical and elderly care facilities (Figure 3).

**Figure 3:**
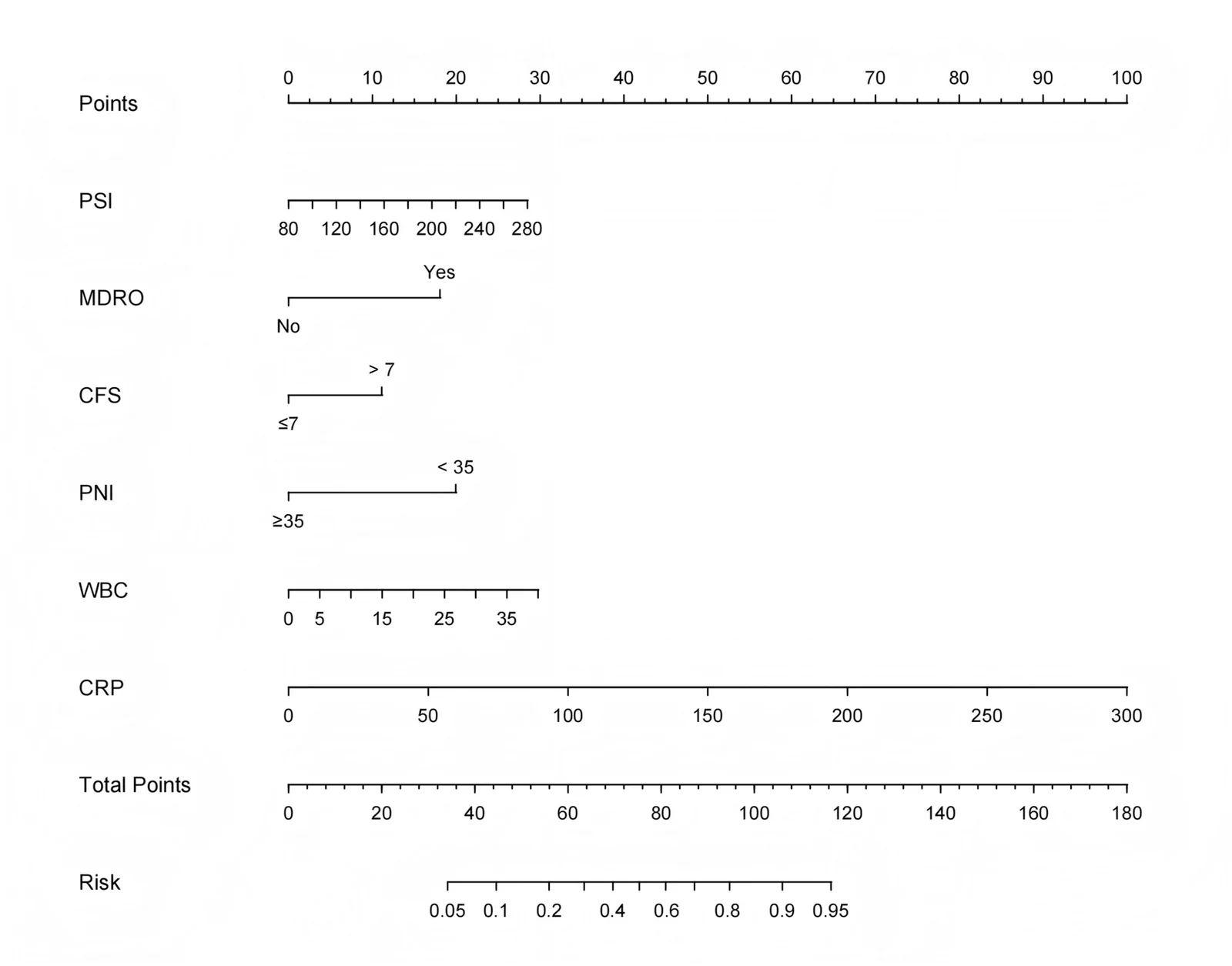
Nomogram for predicting mortality among older patients with pneumonia in integrated medical and elderly care facilities based on the PSI score. MDRO, multi-drug resistant organism; PSI, pneumonia severity index; PNI, prognostic nutritional index; CFS, clinical frailty scale; WBC, white blood cell; CRP, C-reactive protein.

### Evaluation and validation of the prognostic prediction model for mortality in older patients with pneumonia in integrated medical and elderly care facilities

The receiver operating characteristic (ROC) curve demonstrated that the nomogram model yielded an area under the curve (AUC) of 0.889 (95% CI: 0.843–0.938) for predicting mortality risk among older patients with pneumonia in integrated medical and elderly care facilities. The sensitivity was 91.5%, the specificity was 71.4%, the maximum Youden index was 0.629, and the optimal cut-off value was 0.207. The AUC of PSI used alone was 0.699 (95% CI: 0.635–0.764). DeLong test revealed that the discriminative ability of the nomogram was significantly superior to that of the standalone PSI (*P* ≤ 0.05)) (Figure 4). Internal validation was performed via Bootstrap resampling with 1,000 replicates. The optimism-corrected AUC was 0.878, with a slope of 0.9316 and an intercept of −0.0584. The calibration curve showed good agreement between the predicted risks and observed outcomes,The bootstrap-corrected Brier score of this model was 0.100, the apparent Brier score was 0.093, and the baseline Brier score was 0.188, with a Brier skill score of 0.467. The calibration curve of the nomogram predicting prognosis among elderly patients with pneumonia in integrated medical and elderly care facilitie demonstrated good overlap between the predicted and ideal curves, suggesting good discrimination and calibration (Figure 5). The decision curve analysis (DCA) curve lay mostly above the two extreme reference curves. The nomogram provided positive net benefit within the threshold probability range of 0 to 0.6 (Figure 6).

**Figure 4:**
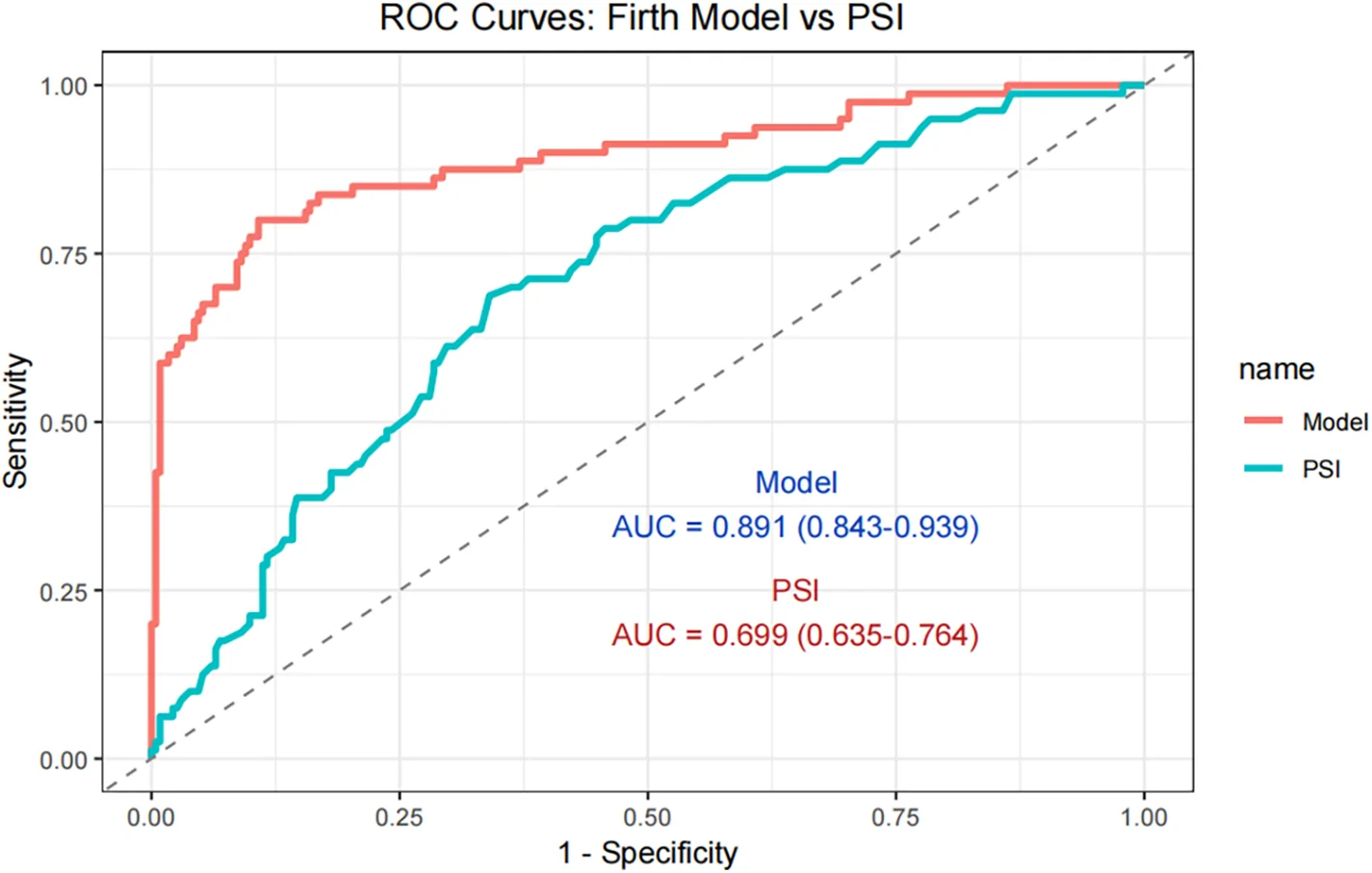
Receiver operating characteristic (ROC) curve of the prognostic model for mortality among older patients with pneumonia in integrated medical and elderly care facilities.

**Figure 5:**
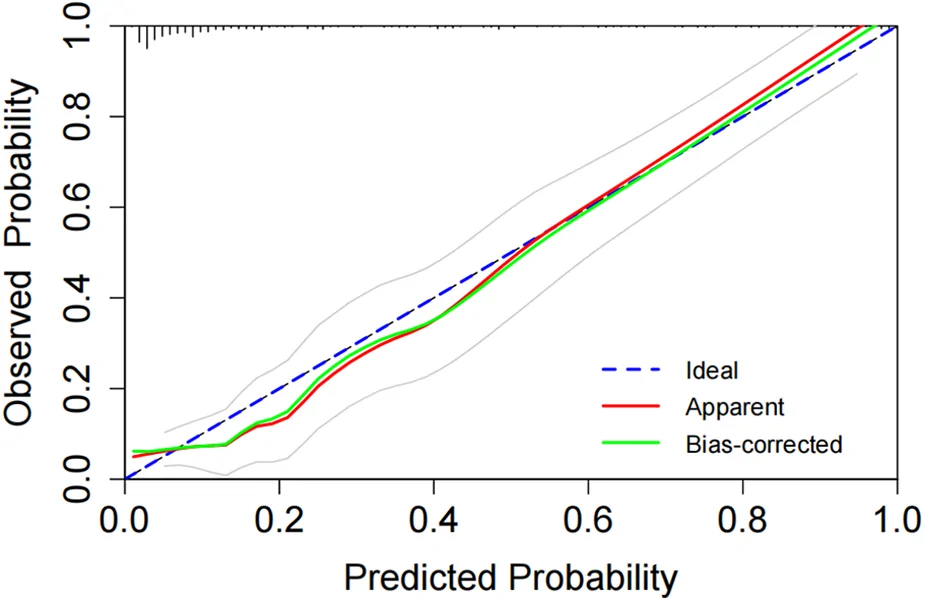
Calibration curve of the prognostic model for mortality among older patients with pneumonia in integrated medical and elderly care facilities.

**Figure 6:**
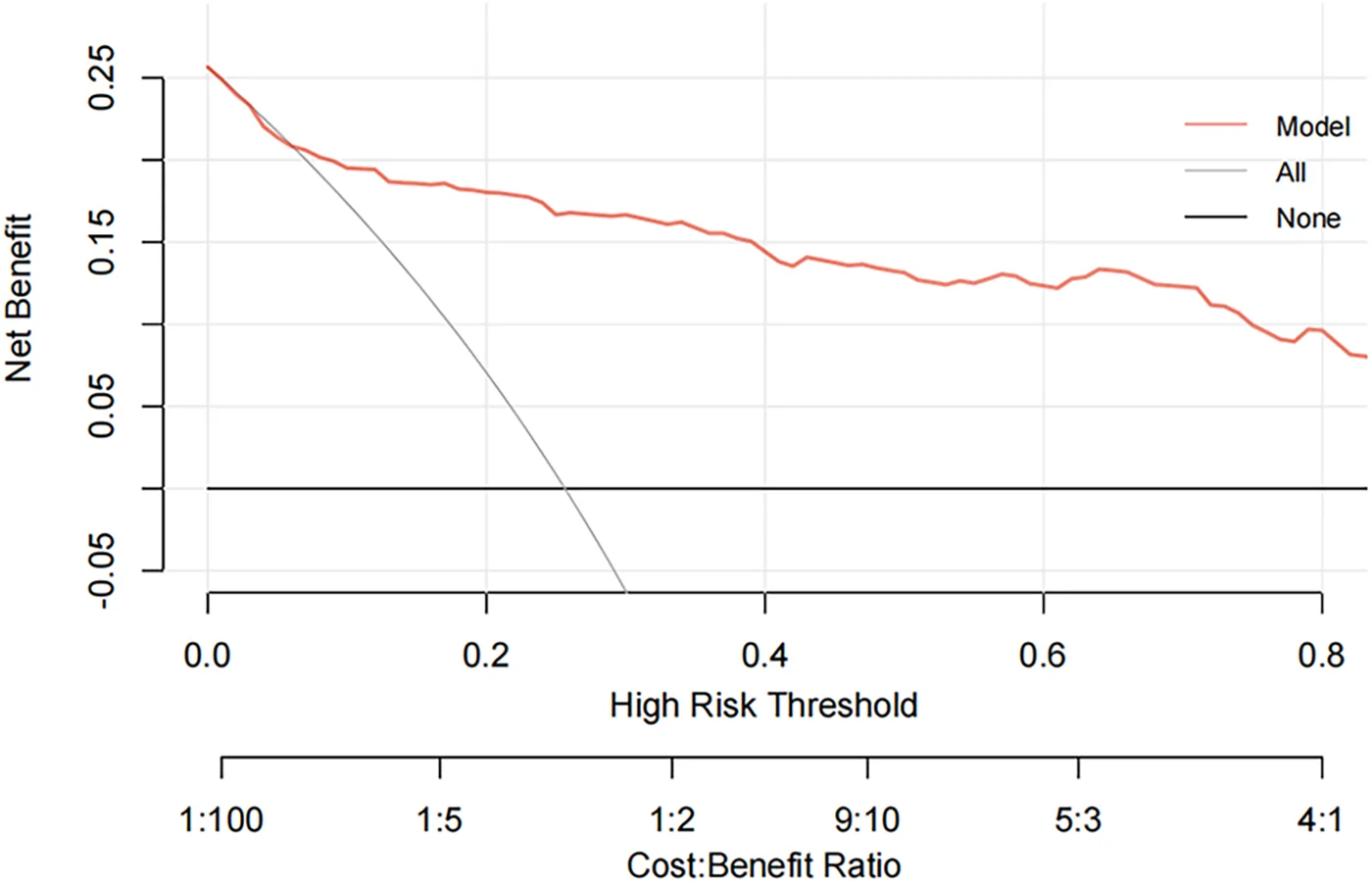
Decision curve analysis (DCA) of the prognostic model for mortality among older patients with pneumonia in integrated medical and elderly care facilities.

## Discussion

### Overview of main findings

With population ageing, the number of bedridden older adults in integrated medical and elderly care facilities continues to rise. Pneumonia in this population carries a high incidence and poor mortality prognosis, highlighting an urgent need for tailored risk-assessment tools (23). This study enrolled 312 older patients with pneumonia from integrated medical and elderly care facilities. A nomogram for mortality prediction was developed by integrating multiple geriatric-specific clinical variables on the basis of the Pneumonia Severity Index (PSI). After variable screening using LASSO regression, six predictors were identified: white blood cell(WBC) count, C-reactive protein (CRP), multidrug-resistant organism（MDRO） infection, PSI score, Clinical Frailty Scale (CFS) > 7, and Prognostic Nutritional Index (PNI) < 35. A visual nomogram was constructed using Firth bias-corrected logistic regression. The original AUC of the model was 0.891, with an optimal cut-off value of 0.719, sensitivity of 80%, and specificity of 89%. After internal validation via 1,000 Bootstrap resamples, the optimism-corrected AUC reached 0.878. Calibration curves demonstrated good agreement between model-predicted probabilities and observed mortality outcomes. Decision curve analysis (DCA) revealed stable clinical net benefit, indicating favourable predictive performance for mortality risk among older patients with pneumonia in integrated medical and elderly care facilities.

### Comparison with previous studies

The PSI is a classic tool for assessing severity and prognosis in community-acquired pneumonia (CAP). Our findings are consistent with the original PSI validation studies; nevertheless, the standalone PSI has notable limitations and is poorly suited for older residents in integrated medical and elderly care facilities, who are characterised by advanced age, multimorbidity, malnutrition and physical frailty. The PSI focuses primarily on the severity of pulmonary infection and fails to comprehensively capture systemic physiological reserve and pathogen-related features (24). Most previously reported prognostic models for pneumonia in older adults were developed for community-dwelling or hospitalised older CAP patients, with few studies targeting pneumonia acquired in integrated medical and elderly care institutions. Considering the disease characteristics of this specific population, we constructed a multidimensional combined prognostic model incorporating inflammatory, nutritional, frailty and multidrug-resistance infection markers on top of the PSI. Compared with the standalone PSI (AUC = 0.699), our model achieved substantially improved predictive performance. It addresses the insufficient accuracy and limited coverage of traditional pneumonia severity scores and fills the research gap in mortality prediction models tailored for older adults with pneumonia in integrated medical and elderly care settings.

### Interpretation of joint effects of multiple predictors

As the core baseline component of our model, the PSI integrates age, comorbidities, vital signs, laboratory and imaging variables to stratify baseline pneumonia severity and represents a well-established framework for mortality risk stratification. However, this scoring system inadequately captures risk factors specific to institutionalised older adults, including frailty, nutritional deficiency and drug-resistant infection. White blood cell count (WBC) is a fundamental marker of innate immune activation following bacterial infection and reflects the mobilisation of peripheral inflammatory cells (25). In frail older adults, immune responses may be blunted, so the magnitude of WBC elevation does not always correlate with infection severity; therefore, WBC should be interpreted together with CRP (26). CRP is an acute-phase reactant that rises rapidly after infection and quantifies systemic inflammatory burden dynamically. It compensates for the static nature of the PSI, which cannot capture progressive systemic inflammatory injury (27). Persistently elevated CRP indicates infection-triggered systemic inflammatory response syndrome and markedly increases the risk of organ failure and death (28). Multidrug-resistant organism infection represents a population-specific high-risk factor in our cohort. Resistant pathogens severely limit the choice of initial empirical antimicrobial therapy, increasing the risk of treatment failure and persistent infection (29). Furthermore, multidrug-resistant infections are often associated with longer hospital stays and higher inflammatory burden, further compromising the fragile physiological reserve of older patients and worsening mortality outcomes (30). A CFS >7 indicates severe physical frailty, reflecting depletion of physiological reserve across multiple organ systems and reduced compensatory capacity (31). Such patients are vulnerable to rapid decompensation of multiple organs under infectious stress and poorly tolerate the insult of pneumonia, making frailty a powerful mortality predictor in older populations (32). A PNI <35 indicates moderate to severe malnutrition. The Prognostic Nutritional Index combines serum albumin and lymphocyte counts to simultaneously reflect nutritional reserve and cellular immune function (33). Low PNI impairs the capacity to engulf and clear pathogens, hindering infection control. Malnutrition may synergise with infection, drug resistance and frailty to amplify the risk of adverse outcomes (34). Collectively, these six variables complement one another and exert synergistic risk effects, enabling comprehensive evaluation of mortality risk factors in older adults with pneumonia receiving integrated medical and elderly care. This underlies the superior predictive performance of our combined model relative to the standalone PSI.

### Clinical implications

Tailored to older patients with pneumonia in integrated medical and elderly care facilities, this model overcomes the weaknesses of conventional pneumonia severity assessment tools and supports clinical decision-making in multiple scenarios. First, triage decisions: the model enables rapid mortality risk stratification into high-, intermediate- and low-risk subgroups, helping clinicians judge whether referral to a higher-level hospital or enhanced monitoring is required and optimising allocation of healthcare resources. Second, monitoring intensity: intensified surveillance of vital signs, oxygen saturation and consciousness can be implemented for patients classified as high-risk, whereas routine monitoring may be sufficient for low-risk patients to reduce unnecessary healthcare expenditure. Third, infection control interventions: for individuals at high risk of multidrug-resistant infection, contact precautions and environmental disinfection can be initiated early to block cross-transmission of resistant organisms. Fourth, antimicrobial stewardship: for patients with high inflammatory burden and high risk of multidrug-resistant infection, early pathogen sampling and individualised anti-infective regimens can be prescribed to facilitate refined antimicrobial management and reduce overuse of broad-spectrum antibiotics. Fifth, family communication: quantitative mortality risk estimates generated by the model provide objective evidence for shared decision-making, helping families understand disease severity and improving treatment adherence. The outputs may also inform palliative care planning for patients at the end of life.

### Strengths of the present study

This study has good innovativeness and clinical value. First, the cohort is well-defined: we focused on the understudied population of older patients with pneumonia in integrated medical and elderly care facilities and developed a dedicated prognostic model to fill the relevant research gap. Second, the statistical approach is rigorous. LASSO regression was used to handle collinearity among predictors, and Firth bias correction was applied to reduce statistical bias in this retrospective study, ensuring robust model development. Third, the assessment framework is comprehensive. Incorporating geriatric-specific markers of inflammation, nutrition, frailty and multidrug-resistant infection, the model transcends the limitations of traditional standalone pneumonia scoring systems. Fourth, the validation strategy is comprehensive. ROC analysis, calibration curves, DCA and Bootstrap internal validation were used to verify stable performance across discrimination, calibration and clinical net benefit. Fifth, the model is clinically practical. The visual nomogram enables rapid bedside risk estimation without complex calculations, which is suitable for primary care and integrated medical and elderly care institutions and has promising scalability. The development, validation and reporting of this prediction model strictly followed the TRIPOD statement (Type 1).

### Limitations

This study has several limitations. First, it was a single-centre retrospective cohort study with a relatively restricted sample source. Inherent to retrospective designs, selection bias and information bias are unavoidable. Inaccuracies in medical records may also confound the results. Second, although Firth correction and Bootstrap internal validation were used to improve model accuracy and reduce statistical bias, overfitting cannot be fully excluded. The predictive performance for patients with complex comorbidities or extremely critical illness remains to be optimised. In addition, we only examined short-term in-hospital mortality and did not conduct time-to-event survival analysis; therefore, the dynamic long-term prognostic effects of each predictor could not be characterised. Only internal validation was performed, and external cohort data are lacking to support the generalisability of the model. The study population comprised older adults from domestic integrated medical and elderly care facilities, so the findings may not be directly applicable to populations in other healthcare systems or geographic regions. Regarding data processing, complete case analysis was adopted for model building. Given the low overall missing rate and approximately random missing pattern, multiple imputation was not performed to simplify model interpretation, which represents a methodological limitation.

### Future perspectives

To address these limitations, future multicentre, large-sample prospective cohort studies are warranted to expand the sample pool, conduct external validation and further evaluate and improve the generalisability of the model. Further analyses of variable interactions and subgroup stratification may help streamline the predictor set, improving model efficiency and variable independence. Time-to-event survival analyses can be introduced to explore the dynamic long-term prognostic impacts of predictors and enhance the model's capacity for long-term outcome assessment. The model may be optimised using intelligent algorithms to develop simple mobile assessment tools and streamline bedside workflows. Prospective interventional studies are also needed to verify the real-world clinical benefits of risk-stratified care, including individualised monitoring, targeted anti-infective therapy and stratified referral guided by this model. Ultimately, this work aims to generate high-quality evidence to support individualised, standardised management of older patients with pneumonia in integrated medical and elderly care facilities.

## Conclusion

This study focused on high-risk older patients with pneumonia in integrated medical and elderly care facilities, a population characterised by advanced age, multimorbidity, frailty, malnutrition and a high prevalence of multidrug-resistant infections. We constructed a multidimensional prognostic prediction model incorporating the PSI, inflammatory markers, frailty, nutritional indices and aetiological factors. The model outperformed the standalone PSI and can be used for mortality risk stratification, individualised monitoring, infection control and refined antimicrobial stewardship in this patient group, providing evidence to support precision care and prognostic assessment for pneumonia in integrated medical and elderly care settings. This is a single-centre retrospective study with limitations including restricted sampling, absence of external validation, lack of long-term outcome assessment and inherent biases of retrospective research. Future multicentre large prospective studies will perform external validation, integrate long-term survival analysis and artificial-intelligence-based optimisation to further explore clinical utility, refine the dedicated prognostic assessment system for older adults in integrated medical and elderly care facilities, facilitate clinical management and improve patient outcomes.

## Data Availability

The original contributions presented in the study are included in the article/Supplementary Material, further inquiries can be directed to the corresponding author.
